# Compound words prompt arbitrary semantic associations in conceptual memory

**DOI:** 10.3389/fpsyg.2014.00222

**Published:** 2014-03-14

**Authors:** Bastien Boutonnet, Rhonda McClain, Guillaume Thierry

**Affiliations:** ^1^ESRC Bilingualism Centre, Bangor UniversityBangor, UK; ^2^School of Psychology, Bangor UniversityBangor, UK; ^3^Department of Psychology, Penn State UniversityPA, USA

**Keywords:** semantics, concepts, compound-words, linguistic relativity, ERPs

## Abstract

Linguistic relativity theory has received empirical support in domains such as color perception and object categorization. It is unknown, however, whether relations between words idiosyncratic to language impact non-verbal representations and conceptualizations. For instance, would one consider the concepts of *horse* and *sea* as related were it not for the existence of the compound *seahorse*? Here, we investigated such arbitrary conceptual relationships using a non-linguistic picture relatedness task in participants undergoing event-related brain potential recordings. Picture pairs arbitrarily related because of a compound and presented in the compound order elicited N400 amplitudes similar to unrelated pairs. Surprisingly, however, pictures presented in the reverse order (as in the sequence *horse*–*sea*) reduced N400 amplitudes significantly, demonstrating the existence of a link in memory between these two concepts otherwise unrelated. These results break new ground in the domain of linguistic relativity by revealing predicted semantic associations driven by lexical relations intrinsic to language.

## Introduction

The Whorfian hypothesis that language may influence other cognitive processes has recently become a major topic in psycholinguistics and neuroscience, probably because evidence in this area is directly informative as regards long-standing debates on language encapsulation (Fodor, 1975, 2008; Chomsky, 2000).

Over the past two decades, the Whorfian hypothesis has undergone a significant revival. First, and in agreement with early criticism of the hypothesis, a deterministic view of linguistic relativity has been dismissed. If anything, in light of compelling evidence that high-level cognitive operations are possible without language, this position becomes untenable (Gallistel, 1989; Feigenson et al., 2004; Gordon, 2004). Subsequent developments of the hypothesis have lead to a non-deterministic account according to which language influences thought without necessarily determining it. One recent theoretical development is the *label-feedback* hypothesis (Lupyan, 2012), which proposes that language is highly interconnected with other cognitive processes such as categorization and that it produces transient modulations of on-going neural processing at different functional levels.

Recent studies have highlighted areas where lexical and grammatical information affect domain-general cognitive processes. For example, color terminology has been shown to influence categorical perception of color in monolingual and bilingual speakers (Gilbert et al., 2006; Thierry and Wu, 2007; Franklin et al., 2008; Roberson et al., 2008; Athanasopoulos et al., 2010; Liu et al., 2010). Similarly, linguistic labels for more visually complex objects have also been shown to influence categorical perception behaviorally [cats vs. dogs (Gilbert et al., 2008); or symbols vs. digits (Lupyan and Spivey, 2008)] and neurophysiologically [cups vs. mugs (Boutonnet et al., 2013)]. Language-specific lexicalization of events and spatial representation has also been shown to affect speakers' perception, recollection, and even gaze patterns when exploring pictures and videos depicting events (Bowermann and Choi, 1991; Majid et al., 2004; Flecken, 2010; Papafragou and Selimis, 2010). Finally, several studies have provided evidence that grammatical number and gender expression can alter speakers' object perception and categorization (Boroditsky et al., 2003; Saalbach and Imai, 2007; Athanasopoulos and Kasai, 2008; Cubelli et al., 2011; Boutonnet et al., 2012).

Despite accumulating evidence in favor of linguistic relativity, critics remain unconvinced. One of the greatest limitations of most previous studies on the hypothesis is their heavy reliance of behavioral measures. Behavioral evidence leaves open the possibility of a contamination by explicit top-down strategies soliciting language processing during tasks that are misconstrued as non-verbal, which, in the context of an investigation of unconscious and automatic effects of language on other cognitive processes, is insufficient, and could boil down to a mere effect of language on language [“thinking for speaking” (Slobin, 2003) see also, (cf. Pinker, 2007)].

While most of the literature on linguistic relativity has focused on effects at the interface between language and other cognitive processes, here, for the first time, we study idiosyncratic relations existing within language and test whether arbitrary relations between lexical entities induce arbitrary associations in semantic memory. We tested whether the existence of compound words lead to associations between the concepts referred to by their constituents (e.g., between *sea* and *horse* in *seahorse*). While most of the current knowledge in the domain of compound processing comes from studies involving written compound words, we decided to use pictorial stimuli in order to avoid direct access to language since the study aims to tap into conceptual memory. Several studies on compound processing have provided evidence for a dual-route access, where compounds are both processed as whole words and decomposed into constituents during reading. Such a model is supported by effects of compound morpheme frequency in studies of eye movements (Andrews et al., 2004; Hyönä et al., 2004), ERPs in the auditory modality (Koester et al., 2007) and behavioral priming (Duñabeitia et al., 2008) recorded in reading tasks. In addition, several neuroimaging studies have identified combinatorial processes involved in comprehension of simple noun-noun (Graves et al., 2010) and metaphorical phrases (Forgács et al., 2012). Finally, Gagné and Spalding (2009) have suggested that the integration of compounds may rely on the same combinatorial processes as mentioned above, which involve both psycholinguistic and conceptual knowledge.

The bulk of studies in favor of a decompositional account suggest that compound constituents do not make equal contributions to the meaning of the whole. According to Libben et al.'s (2003) classification there are three types of compound words: those made of two semantically transparent constituents as in *bedroom*, those made of a mixture of a transparent and opaque constituents (e.g., *strawberry* or *jailbird*) or two semantically opaque constituents (e.g., *hogwash*). Indeed, opaque constituents lead to slower compound processing especially when that opaque constituent is the head of the compound (see below).

The internal order and relationship between the constituents of a compound, often referred to as *headedness*, has also been shown to play a critical role in compound processing. In English, most compound words are right-headed, meaning that the final constituent carries most of the compound's meaning, whereas the initial constituent acts as a modifier—akin to the relationship between an adjective and a noun in the noun-phrase. In the compound *teapot*, for instance, the second constituent (*pot*) provides the core concept while the initial constituent (*tea*) specifies what the pot is for. Studies of priming between a compound word and its constituents show that the head constituent reliably improves participants' detection rates. In addition, headedness has been shown to compensate a lack in semantic transparency (Jarema et al., 1999) as well as to overcome a processing deficit in neglect dyslexia, where patients with left-neglect read left-headed compounds better than right-headed ones (Semenza et al., 2011). Finally, an ERP study of Italian compound words demonstrated a modulation of the P300 wave in relation to head position, indicating a fundamental role of head position in compound processing (El Yagoubi et al., 2008). Similar evidence highlighting the role of the head component in compound access comes from a study by Isel et al. (2003) conducted in the auditory domain where the prosodic structure of the first constituent in German compounds appears to be crucial in triggering the decompositional route.

The studies reviewed so far provide solid evidence that compounds are most likely decomposed in reading and hints that they are not represented as a whole unit in the mental lexicon—with the exception of compounds made of exclusively semantically opaque constituents. However, the issue of decomposition during compound word processing remains debated and theoretical standpoints tend to vary with input modalities. Indeed, Kuperman et al. (2008) and Janssen et al. (2008) argue that whole-word processing or whole-word representation is still possible for all kinds of compound words while Blanken (2000) provides evidence in favor of compound decomposition even in the case of strictly opaque compounds.

Additionally, while the studies reviewed above essentially focus on the semantic roles of compounds' constituents and have outlined different degrees of priming depending on factors such as headedness and transparency, a recent study by Koester et al. (2007), in the auditory domain, disentangles possible effects of semantic processing from morphological effects on compound decomposition by manipulating constituent transparency and gender congruency. In this study, purely morphological combinatorial processes modulated the LAN ERP component while semantic (re-)composition modulated N400 amplitudes. This evidence reinforces the idea that the meaning of compound words is likely composed online.

Altogether the studies on compound processing as well as studies investigating their representation strongly support an account of compound words involving decomposition and then re-composition. In an attempt to integrate the majority of the results in favor of decomposition, Libben (1998) puts forward a model of compound representation articulated around three levels: stimulus level, lexical level, and conceptual level. Each representation level contains information about the structural links between the constituents of compound words. The stimulus level is said to contain information about the shape of compound words which enable speakers of English, for example, to identify the word “redberry” as a compound although such a lexical entry does not exist in English. The lexical level is said to represent word forms (i.e., “legal” compound entries such as “strawberry” or “blueberry” vs. entries such as “redberry”) and the morphological relationship between the compound word's constituents (i.e., whether the compound “strawberry” contains both lexical entries “straw” and “berry” or just “berry” unlike the compound word “blueberry”). Finally, the conceptual level is thought to represent the contribution of the constituents of a compound toward the meaning of the compound word (i.e., whether the meaning of “blueberry” is made from the combination of the meanings of “blue” and “berry” or of only one of the constituents'—“strawberry”).

In this study, we hypothesize that the combinatorial processes at the morphological level and at the semantic level, and possibly the type of relationships proposed in Libben's (1998) account, should lead to the emergence of links between the constituents of compound words. In other words, because *sea* and *horse* are in the same compound (*seahorse*) and have to be accessed separately and recombined, a “linked-to” feature in the representation of the individual words themselves might emerge. If this is the case, we expect constituents to be able to prime each other. In the ERP literature, a large number of publications converge in predicting a reduction of the N400 for pictures conceptually related to a picture prime (e.g., a picture of a *fish* following that of the *sea*) compared to that elicited by unrelated pictures (Barrett and Rugg, 1990; Ganis et al., 1996; Kutas and Federmeier, 2011). Critically, we expected the lexical association within a compound word linking two picture names to induce a conceptual link, which would in turn reduce the amplitude of the N400. In order to tease apart the lexical association from the conceptual link contribution to N400 reduction, we presented the pictures in the reverse order of that found in compounds. In this latter case, we had no prediction regarding potential N400 modulations.

## Materials and methods

### Participants

Participants were 16 (nine female, seven male, age: 21.9 ± 0.9) native speakers of English and students of the School of Psychology at Bangor University. They were offered course credits for their participation in the study that was approved by the local ethics committee.

### Materials

We selected 51 compound words (e.g., sandcastle) and a prototypical picture for each constituent word embedded within them (e.g., a picture for sand, and one for castle). Altogether, 102 highly recognizable photographs were selected from online image databases. The pictures were arranged into four fully rotated experimental conditions: semantically related (Related), related via a compound and in the compound order (Compound), related via a compound but in the reverse order (Reversed), and semantically unrelated (Unrelated). Only stimuli presented in the Related condition were coded as related and stimuli presented in the Compound and Reversed conditions were coded as unrelated. Picture stimuli subtending approximately 10° of visual angle were presented on a white background in the center of a 19” CRT monitor.

Association strength of all picture name pairs was extracted from the Edinburgh Associative Thesaurus (Kiss et al., 1973) and a repeated-measures Analysis of Variance (ANOVA) was conducted to test for differences in semantic relatedness between picture names across conditions (see Table 1). There was a significant main effect of condition [*F*_(3, 147)_ = 9.6, *p* < 0.0001, η^2^_*p*_ = 0.17]. *Post-hoc* comparisons showed that the Related and Compound conditions were significantly more related than the Reversed and Unrelated conditions (Bonferroni, *p*s < 0.05), respectively, but did not differ significantly from each other. Critically, the Reversed pairs were not significantly more related than the Unrelated pairs (Bonferroni, *p* > 0.1).

**Table 1:** **Experimental conditions, example of names of pictures in each of the conditions, and Edinburgh Associative Thesaurus (Kiss et al., 1973) mean relatedness scores**.

**Experimental condition**	**Picture names**		**Association strength**
Related	Sea	Fish	0.036 (0.009)
Compound	Sea	Horse	0.045 (0.008)
Reversed	Horse	Sea	0.008 (0.003)
Unrelated	Sea	Cake	0.003 (0.002)

*s.e.m. is given in brackets*.

Since the 51 picture pairs associated with the compound stimuli were fully rotated across experimental conditions, the constituent words had to refer to highly imageable and familiar objects whilst belonging to common compound words and enabling the construction of related and unrelated lists independently of within compound relationships (see Supplementary Material for the full stimuli list). It was therefore impossible to select compound words from one homogenous semantic transparency category. This lead to a stimulus list comprised of 70% Transparent-Transparent, 10% Transparent-Opaque, 16% Opaque-Transparent, and 4% Opaque-Opaque compounds.

### Procedure

Participants signed a consent form to take part in the study that was approved by the Ethics Committee of Bangor University. They were tested individually in a quiet room and instructed to press a given button when two consecutive pictures were related and another button when they were unrelated. Participants were not informed about the presence of pairs derived from compound words and were instructed to focus on evaluating the conceptual relatedness of the pictures. On each trial, a fixation cross was presented for 250 ms, followed immediately by the first picture for a duration of 500 ms, then the second picture appeared after a random variable inter-stimulus interval of 400, 450, 500, 550, 600 ms, averaging to 500 ms, and remained on screen for a duration of 3000 ms maximum or disappeared upon participant response. A blank screen with a duration of 500 ms on average separated each trial, in order to cancel offset effects. Block order was fully counterbalanced across participants and stimulus presentation was fully randomized.

### Electrophysiological recording

The EEG was continuously recorded at a rate of 1 kHz from 64 Ag/AgCl electrodes placed according to the extended 10–20 convention. Two additional electrodes were attached above and below the left eye and on either side of the left and right eye in order to monitor for eye-blinks and horizontal eye movements. Cz was the reference electrode during acquisition. Impedances were maintained below 5 kΩ for all 64 electrodes and below 10 kΩ for vertical electrooculogram electrodes. EEG activity was filtered offline with a high-pass 0.1 Hz filter (slope 12 dB/oct) and a low-pass 30 Hz filter (slope 48 dB/oct).

### Behavioral data analysis

Two separate repeated measures ANOVAs were carried out on RT and accuracy with the four conditions (related, compound, reversed, and unrelated) as within-subject factors.

### Electrophysiological data analysis

Eye-blink artifacts were mathematically corrected using the algorithm provided in Scan 4.4™. The algorithm is derived from the method advocated by Gratton et al. (1983). Note that eye-blinks occurred mostly after the response was made as a consequence of special instruction given to the participants. ERPs were then computed by averaging EEG epochs ranging from −100 to 1000 ms after stimuli onset. Baseline correction was applied in relation to 100 ms of pre-stimulus activity and individual averages were re-referenced to the common average of all scalp-electrodes. ERPs time-locked to the onset of target pictures were visually inspected and mean amplitudes were measured in temporal windows determined based on variations of the mean global field power (Picton et al., 2000). Three components were identified as expected. The P1 and N1 were maximal at parietal sites and were measured in the 100–150 ms range for the P1 and 170–230 ms for the N1. The N400 was maximal over central sites and was measured in the 350–480 ms window. Peak latencies were measured at sites of maximal amplitude (PO8 for the P1 and N1, Cz for the N4) and mean ERP amplitudes were measured in regions of interest around the sites of maximal amplitude (O1, PO3, PO7, O2, PO4, PO8 for the P1 and N1; F3, Fz, F4, FC1, FCz, FC2, C1, Cz, C2 for the N4). Note that we did not conduct a full-scalp analysis because the modulation of the ERP components were predicted to occur in the regions of interest and therefore statistical analyses of ERP mean amplitude were conducted in sets of electrode determined a priori. Finally, mean amplitudes were subjected to a repeated-measures ANOVA with condition (Compound, Reversed, Related, and Unrelated), anteriority (anterior, central, posterior) and laterality (left, center, right) as within-subject factors.

## Results

### Behavioral data

**Figure 1:**
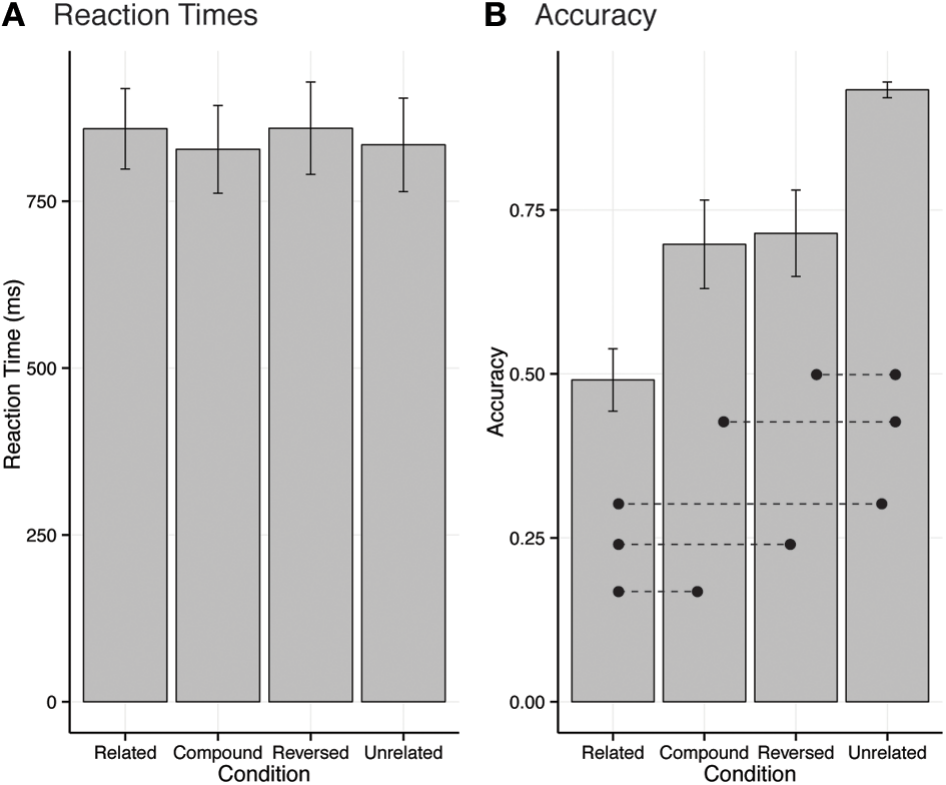
**Plot of mean reaction times (RTs) and accuracy in the four experimental conditions**. Bars depict RTs, dots depict accuracy. Error bars depict s.e.m. Dotted lines depict significant differences between conditions. **(A)** Reaction times for each condition. **(B)** Accuracy for each condition.

Statistical analyses carried out on RTs revealed no significant differences between experimental conditions [*F*_(3, 45)_ = 2.12, *p* > 0.05, η^2^_*p*_ = 0.03; Figure 1A]. Accuracy analysis revealed a main effect of condition [*F*_(3, 45)_ = 17.17, *p* < 0.001, η^2^_*p*_ = 0.37]. *Post-hoc* found no significant differences between the Compound and Reversed conditions (Bonferroni, *p* > 0.05), but the Related and Unrelated conditions were significantly different from all other conditions, with Related leading to significantly lower accuracy scores than all other conditions (*M* = 0.53 ± 0.03, Bonferroni, *p* < 0.05) and Unrelated leading to significantly higher accuracy scores than all other conditions (*M* = 0.92 ± 0.02, Bonferroni, *p* < 0.05), as illustrated in Figure 1B. Finally, accuracy significantly differed from chance (0.5) in all conditions (*ps* < 0.0001) but the Related condition.

### Electrophysiological data

**Figure 2:**
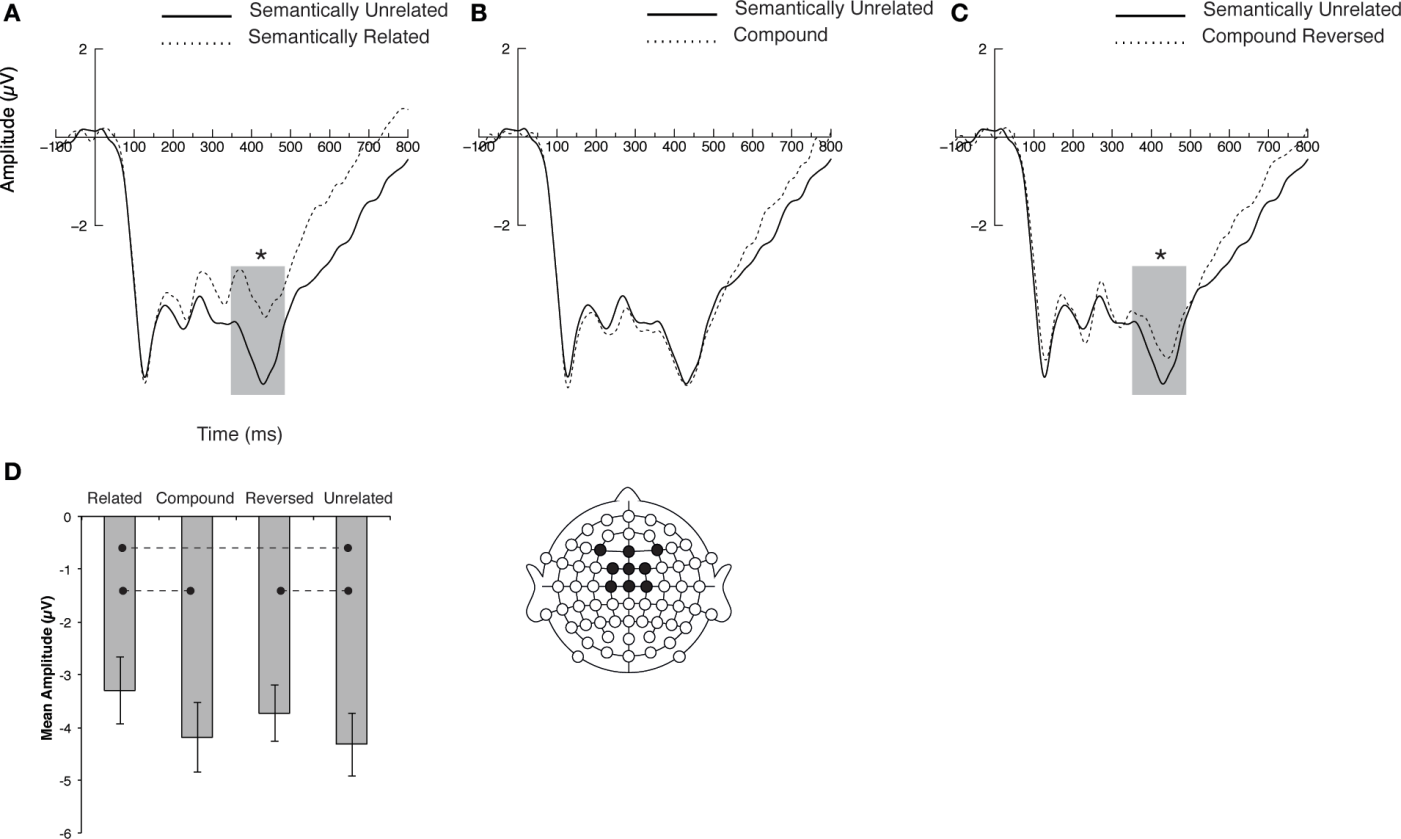
**Event-related brain potentials elicited by the four experimental pairs averaged across blocks. (A)** ERPs elicited in the Related and Unrelated conditions. **(B)** ERPs elicited in Related and Compound conditions. **(C)** ERPs elicited in the Reversed and Unrelated conditions. **(D)** Bar plot of mean N400 amplitudes in all experimental conditions. Waveforms correspond to linear derivations of electrodes F3, Fz, F4, FC1, FCz, FC2, C1, Cz, C2. Error bars depict s.e.m. Dotted lines on bar plot depict significant differences between conditions. ^*^ is used to denote significant differences.

Statistical analyses carried out on N400 mean amplitudes revealed a main effect of condition [*F*_(3, 45)_ = 6.84, *p* < 0.001, η^2^_*p*_ = 0.31], a main effect of anteriority [*F*_(2, 30)_ = 23.6, *p* < 0.001, η^2^_*p*_ = 0.61], and a main effect of laterality [*F*_(2, 30)_ = 8.91, *p* < 0.001, η^2^_*p*_ = 0.37]. There was no significant interaction. *Post-hoc* paired *t*-tests revealed significant differences between Related and Unrelated [*t*_(15)_ = −3.04, *p* < 0.05; Figure 2A], between Related and Compound [*t*_(15)_ = −3.6, *p* < 0.05] and between Reversed and Unrelated [*t*_(15)_ = 2.3, *p* < 0.05; Figure 2C]. Unrelated and Compound conditions did not differ (*p* > 0.05; Figure 2B. The mean amplitude of each condition is summarized in Figure 2D.

## Discussion

This study investigated whether a phenomenon such as word compounding, which leads to an artificial boost of lexical relatedness idiosyncratic to language, has consequences regarding the organization of conceptual-semantic knowledge. Whereas, ERPs elicited by pictures related because of the existence of a compound in the lexicon failed to reduce N400 ERP amplitudes, the same pictures presented in the reverse order significantly reduced N400 amplitudes as compared to semantically unrelated ones.

Although there was no difference in RTs between conditions, there was a significant difference in accuracy such that conceptually related pictures led to error rates not different from chance. Judging the relatedness of pictures is not a difficult task *per se* but this result is not surprising given that the related pictures were not prototypically related (cf. Table 1). This was due to the fact that the experimenters were not at liberty to select any related picture pair to serve as related pairs because this would have engendered uncontrolled differences in low-level visual differences, visual complexity familiarity, prototypicality, etc., which are known to have dramatic influences on the timing of some processes (Thierry et al., 2007; Dering et al., 2011). In any case, we can be reassured that semantic priming did take place, since we obtained highly significant N400 differences between the Related and Unrelated conditions, thereby replicating classical N400 effects (e.g., Barrett and Rugg, 1990; Ganis et al., 1996; Kutas and Federmeier, 2011). It is also important to note that the low accuracy scores were only an issue for the Related condition since participants had accuracy scores higher than 70% in all other conditions (cf. Figure 1) and that the critical comparisons involved the compound, reverse, and unrelated conditions. This analysis confirms one of the benefits of collecting electrophysiological data in more constrained experimental designs where behavioral measures lack sensitivity. Moreover, accuracy was intermediate between related and unrelated conditions in the Compound and Reversed conditions, which, in the absence of RT differences, could be interpreted as a sign that Compound and Reversed pairs were less related than related pairs but still more related than unrelated pairs.

Although Compound and Reversed conditions were indistinguishable on the basis of behavioral data, N400 amplitude was only significantly reduced as compared to unrelated pairs for the latter condition. This result suggests that the lexical link between a compound's constituents has consequences in terms of conceptual-semantic memory associations, since pairs of objects *a priori* unrelated like *horse*–*sea* seem easier to integrate than an unrelated pair (e.g., *horse*–*shell*) or the compound-ordered picture pair (e.g., *sea*–*horse)*. This effect cannot be driven by the particular stimuli used in every condition, since the association scores for the Unrelated and Reversed conditions did not differ, and, as critically explained above, the specific pictures used in all the conditions were identical. The most conservative interpretation of this effect is thus that a link in semantic memory has been imposed by idiosyncratic language connections.

Surprisingly, however, the Compound condition, which had association ratings comparable to the related condition, and is, intuitively, the condition in which the lexical link would have been most obvious, failed to reduce N400 amplitude relative to the unrelated condition. An account of this phenomenon could be that, as participants process compound-ordered pairs of pictures, they access in their lexicon the actual compound word whose lexical frequency is necessarily lower than that of the words depicted by the pictures considered individually. This would also be expected to result in greater negativity in the N400 range (Kutas and Federmeier, 2011). In other words participants were faced with interference between lexical processing elicited by the activation of the compound word and conceptual processing of the pictures. An alternative and perhaps more exciting account of this effect could be that the conflict between the meaning of the compounds' morphemes considered separately and that of the compound word considered as a whole prompted a conflict in semantic memory, reflected by an increase in N400 amplitude. The pattern of results reported in the present study are consistent with N400 modulations reported by Koester et al. (2007) upon presentation of compound words although they interpreted the increased negativity as a trace of semantic recomposition (i.e., integration of the compound as a whole). These hypotheses require further testing in the future.

Irrespective of the explanation for a lack of semantic priming in the compound-ordered condition, the effect observed in the reversed-compound condition constitutes strong evidence that lexical relations imposed by language within the lexicon have implications for semantic memory organization. Indeed, the significant reduction of N400 amplitudes when participants processed the second image of a compound pair presented in reversed order demonstrates the existence of a conceptual link that does not exist for similarly unrelated concepts, which are not artificially related via compounding. We believe that our results support Libben's (1998) model's separation between purely lexical levels (stimulus and lexical levels) and the conceptual level and we believe that our effect is likely to be located in Libben's conceptual level. However, while our results provide support for Libben's (1998) level separations, we do not wish to take a position that singles out which of the types of relationships thought to be stored in Libben's (1998) conceptual level drive our effects. In fact, we believe that our relatedness effect emerges from a generalization of all types of relationships across all three of Libben's (1998) levels. We believe our effect would therefore be independent from the order and modality of priming, that it would encode a relationship in the simple (A linked to B) form (where A and B are a compound's constituents) and, finally, that it would apply to all types of compounds (whether made of transparent or opaque constituents or a mixture of both). Such a hypothesis would, however, need to be substantiated by further evidence.

One may wonder why we offer an explanation in terms of conceptual association for the Reversed condition but a lexical explanation for the lack of N400 reduction in the compound condition. As reviewed in the introduction, studies on constituent priming (Jarema et al., 1999; Libben et al., 2003; Semenza et al., 2011), as well as studies of compound access (El Yagoubi et al., 2008) suggest that the morphological head of a compound has a special status. Therefore, one could argue that pairs of pictures presented in reversed compound order presented the head of the compound first, which should have facilitated conceptual access. We believe, however, that this explanation is unlikely for three reasons: (1) our study only required semantic relatedness decisions on pictures and lexical access—if prompted—must have been collateral or indirect; (2) the task targeted conceptual priming between the compound's constituents rather than between the compound word considered as a whole and its constituents; (3) effects of constituent order in compound processing shown previously (Jarema et al., 1999; Libben et al., 2003; El Yagoubi et al., 2008; Semenza et al., 2011) have not targeted semantic priming between constituents and/or targeted the N400 component directly, making comparison of conclusions speculative. In fact, the reason why we offer an explanation in terms of conceptual association is because the Reversed condition is possibly the best condition in which to assess the amount of *conceptual* priming between the two constituents of a compound. Indeed, this condition teases apart the *semantic* relationship between constituents from the *lexical* relationship otherwise confounded in the Compound condition.

In terms of compound processing, we believe that this study is compatible with the bulk of data in favor of a dual-route and decompositional models of compound access and representation (Isel et al., 2003; Hyönä et al., 2004; Vannest et al., 2005; Duñabeitia et al., 2008) as well as studies which have reported neural correlates of re-composition processes (Gagné and Spalding, 2009; Graves et al., 2010). Indeed, for compound constituents to prime each other in any order of priming (compound order or reversed compound order), these constituents have to be represented as separate units and must bear, as part of their semantic features, a link toward other words through which they might be related lexically. The N400 modulation obtained in the Compound condition could be taken to reflect such compositional processes, an interpretation proposed by Koester et al. (2007). Because the study was designed to investigate potential relatedness effects between the constituents of compound words, we are not able to relate our study to the literature in relation to headedness and semantic transparency within compounds and the effect such features might have on compound access. However, we believe that these features could interact with the constituent-constituent priming effect we report here, which will be addressed in future studies.

Finally, while most of the literature in linguistic relativity focuses on specific linguistic features and how this affects some unrelated cognitive processes, this study offers new perspectives for the study of Whorfian effects arising from properties and relations established within language itself. Future studies will investigate how such effects differ between languages since cross-linguistically compounding may rest on different processes which may favor relatedness effects between compound constituents to different degrees.

## Authors contributions

Bastien Boutonnet, designed study, collected and analyzed data, wrote paper. Rhonda McClain, designed study. Guillaume Thierry, designed study, analyzed data, wrote paper.

### Conflict of interest statement

The authors declare that the research was conducted in the absence of any commercial or financial relationships that could be construed as a potential conflict of interest.
