# Supplementary material for: Compound words prompt arbitrary semantic associations in conceptual memory
Source: Front Psychol. 2014 Mar 14;5:222. doi: 10.3389/fpsyg.2014.00222 (PMC3953663; doi:10.3389/fpsyg.2014.00222)
Supplement: Supplementary file 1 [file Presentation1.PDF]

Appendix

Appendix 1. List of experimental stimuli

Related		Compound		Reversed		Unrelated	
Prime	Target	Prime	Target	Prime	Target	Prime	Target
arm	weight	arm	chair	chair	arm	arm	screen
bed	chair	bed	pan	pan	bed	bed	hole
bomb	hole	bomb	shell	shell	bomb	bomb	pot
bottle	pot	bottle	neck	neck	bottle	bottle	dog
butter	cake	butter	fly	fly	butter	butter	boy
cannon	hole	cannon	ball	ball	cannon	cannon	fish
cow	horse	cow	boy	boy	cow	cow	apple
crab	shell	crab	apple	apple	crab	crab	bow
cross	ring	cross	bow	bow	cross	cross	pick
cross	pin	cross	fire	fire	cross	cross	towel
cup	pan	cup	cake	cake	cup	cup	horse
door	gate	door	bell	bell	door	door	bird
drum	bell	drum	stick	stick	drum	drum	casttle
ear	neck	ear	ring	ring	ear	ear	ball
feather	bird	feather	weight	weight	feather	feather	cake
fire	tree	fire	bird	bird	fire	fire	glasses
flag	tower	flag	ship	ship	flag	flag	weight
flower	stick	flower	pot	pot	flower	flower	ship
foot	shoe	foot	ball	ball	foot	foot	bow
gold	pick	gold	fish	fish	gold	gold	tree
hand	head	hand	bag	bag	hand	hand	hole
hand	back	hand	book	book	hand	hand	fly
honey	cake	honey	moon	moon	honey	honey	chair
horse	fly	horse	shoe	shoe	horse	horse	shell
ink	paper	ink	well	well	ink	ink	snake
king	castle	king	pin	pin	king	king	book
man	boy	man	hole	hole	man	man	shoe
olive	cake	olive	tree	tree	olive	olive	head
pan	fish	pan	cake	cake	pan	pan	ring
paper	book	paper	towel	towel	paper	paper	moon
rain	well	rain	bow	bow	rain	rain	shell
rattle	ball	rattle	snake	snake	rattle	rattle	fish
razor	neck	razor	back	back	razor	razor	ball
sand	ball	sand	castle	castle	sand	sand	tower
sand	shell	sand	paper	paper	sand	sand	fire
sea	fish	sea	horse	horse	sea	sea	cake
sea	ship	sea	shell	shell	sea	sea	neck
shell	boat	shell	fish	fish	shell	shell	back
smoke	fire	smoke	screen	screen	smoke	smoke	gate
spear	bow	spear	head	head	spear	spear	pin

sponge	towel	sponge	cake	cake	sponge	sponge	pan
steam	screen	steam	boat	boat	steam	steam	bell
sun	moon	sun	glasses	glasses	sun	sun	paper
sword	bow	sword	fish	fish	sword	sword	bag
tail	dog	tail	gate	gate	tail	tail	well
tooth	apple	tooth	pick	pick	tooth	tooth	stick
turtle	snake	turtle	neck	neck	turtle	turtle	cake
watch	bag	watch	dog	dog	watch	watch	neck
watch	glasses	watch	tower	tower	watch	watch	fish
worm	fish	worm	hole	hole	worm	worm	boat

Appendix 2. Frequencies of compound words and their constituents. Frequency is expressed as Log10 of HAL…

Compound Word	Frequency	First Constituent	Frequency	Second Constituent	Frequency
armchair	6.415	sun	11.213	apple	11.095
bedpan	3.219	arm	9.925	back	12.882
bombshell	5.537	bed	10.353	bag	9.707
bottleneck	6.944	bomb	9.641	ball	10.558
butterfly	7.494	bottle	9.833	bell	10.119
cannonball	6.157	butter	8.832	bird	9.856
cowboy	7.947	cannon	8.933	boat	9.671
crabapple	3.466	cow	8.89	book	12.16
crossbow	6.436	crab	6.888	bow	8.923
crossfire	6.742	cross	10.301	boy	10.665
cupcake	4.369	cup	10.233	cake	8.74
doorbell	6.273	door	10.893	castle	9.361
drumstick	4.344	drum	9.024	chair	9.83
earring	6.033	ear	9.334	dog	10.974
featherweight	4.477	feather	7.768	fire	11.046
firebird	6.815	fire	11.046	fish	10.329
flagship	7.301	flag	9.636	fly	10.289
flowerpot	3.871	flower	8.746	gate	9.719
football	9.786	foot	10.095	glasses	9.053
goldfish	7.009	gold	10.879	head	11.6
handbag	5.328	hand	11.735	hole	10.158
handbook	8.782	honey	9.115	horse	10.08
honeymoon	7.258	horse	10.08	moon	10.332
horseshoe	6.151	ink	8.63	neck	9.605
inkwell	3.584	king	10.87	pan	9.215
kingpin	5.894	man	12.149	paper	11.067
manhole	6.109	olive	7.99	pick	10.721
pancake	6.509	pan	9.215	pin	10.116
rainbow	8.817	paper	11.067	pot	9.485
rattlesnake	5.46	rain	9.787	ring	10.696

razorback	4.174	rattle	7.118	screen	11.221
sandcastle	3.497	razor	8.091	shell	10.523
sandpaper	5.948	sand	9.219	ship	10.845
seahorse	3.951	sea	10.512	shoe	8.558
shellfish	5.583	shell	10.523	snake	8.56
smokescreen	5.72	smoke	9.72	stick	10.25
spearhead	5.485	spear	7.399	towel	8.131
steamboat	6.303	sponge	7.376	tower	10.079
sunglasses	8.14	steam	8.562	tree	10.212
swordfish	5.024	sword	9.726	weight	10.433
tailgate	5.03	tail	9.33	well	13.222
toothpick	5.631	tooth	8.048		
turtleneck	5.273	turtle	8.408		
watchdog	6.875	watch	10.95		
watchtower	6.609	worm	8.321		
wormhole	7.601				
	**5.986**		**9.476**		**10.246**
